# Does mental health limit organizational behavior, or not? A study drawn on resource conservation

**DOI:** 10.3389/fpsyg.2023.1200614

**Published:** 2023-07-20

**Authors:** Aarthi Chandrasantha Singh, Mohanraj Bhuvaneswari

**Affiliations:** Department of Social Sciences, School of Social Sciences and Languages, Vellore Institute of Technology, Vellore, India

**Keywords:** conservation of resources, organizational citizenship behavior, counterproductive work behavior, mental health, stress, depression, anxiety

## Abstract

The current study is rooted in the theory of conservation of resources, exploring the associations between mental health factors namely stress, anxiety and depression with organizational citizenship behavior and counterproductive wok behavior. Data gathered from an Indian automobile sector revealed interconnections between the variables. It was found that stress, anxiety and depression have a positive relationship with OCB and stress and anxiety have negative relationship with CWB. The manuscript further evaluates the results drawing upon conservation of resources theory as we find striking differences between our hypotheses and results, and that opens up new avenues for future research.

## Introduction

Mental health and behavior are intimately intertwined; any change in mental health will be blatantly apparent in a person’s behavior. According to the World Health Organization (WHO), mental health encompasses not only disability, but also an individual’s subjective well-being, competence, achievement of one’s intellectual and emotional potential, etc., implying that anyone can be mentally affected, but this does not necessitate a clinical diagnosis. It could be due to short-term stress, family concerns, professional issues, or anything else that the individual finds stressful. WHO has highlighted depression, substance addiction, anxiety disorders, and work-related stress as the most prevalent mental health issues in the workplace, resulting in increased absenteeism and decreased productivity. Absenteeism and productivity are indicators of organizational behaviors such as citizenship behavior (productivity) and unproductive work behavior (absenteeism). The levels of a person’s stress, anxiety, and depression have a substantial impact on the organizational performance of an employee; the lower these factors, the greater will be the individual’s performance, which in turn can lead to desired performance of organizational citizenship behaviors (OCB) and reduce the undesirable counterproductive work behavior (CWB). Conservation of Resources (COR) theory is one such theory which is widely used in organizational behavior literature world to understand the employee work behavior, it is basically a stress-motivation theory. In the recent years many researchers have used this theory to understand the relationship between OCB and CWB using many factors some of them related to mental health like psychological contract (Zhong et al., 2021), hindrance stressor (McLarty et al., 2021), job stress (De Clercq and Belausteguigoitia, 2020b), emotional exhaustion (Montani and Dagenais-Desmarais, 2018; Loh and Saleh, 2022; Madhan et al., 2022), fatigue (Xu et al., 2021), and many more. The fact that organizational behavior world has widely studied topics like work stress, psychological capital, emotions at work but studies concerning the clinical mental health aspect like stress, anxiety, and depression at work are highly limited. Thus, we have tried to capture the impact of these mental health factors in our paper affecting the work behaviors, precisely OCB and CWB. Also, the paper tries to understand the psychological deliverables of an individuals in an organization (Sia, 2022).

### Organizational citizenship behavior

Organ (1988) defined organizational citizenship behavior (OCB) as ‘individual behavior that is discretionary, not directly or explicitly recognized by the formal rewards system, and that in the aggregate promotes the effective functioning of the organization’. These behaviors incorporate many dimensions like, sportsmanship, altruism, civic virtue, conscientiousness and courtesy (Organ, 1988). It has been observed that OCB improves employees’ work motivation, performance, and ultimately their career success (Borman and Motowidlo, 1993; Russo et al., 2014; De Clercq and Belausteguigoitia, 2020a). Yet, devoting a substantial amount of energy on volunteering activity can also pose considerable obstacles for employees and conflict with their normal job duties. In other words, when employees accept additional obligations that are not strictly required of them, the resulting distractions may hinder their ability to achieve their allocated performance goals (Culbertson and Mills, 2011; Bolino et al., 2015). When faced with hardship in the office, employees struggle with their organizational functioning and develop anxieties about their future career possibilities, which may express as job stress, or a feeling of being overwhelmed or overburdened by the current environment (Hon and Chan, 2013; Perko et al., 2017; Abdelmoteleb, 2018). Despite the widespread recognition that exposure to stress-inducing work conditions can dissuade employees from engaging in OCB (Cheung and Cheung, 2013; Pooja et al., 2016; De Clercq and Belausteguigoitia, 2020b), there is a paucity of research that explicitly links the experience of mental health to voluntary work efforts, specifically stress, anxiety, and depression following the covid scenario.

Research on organizational citizenship practices has become increasingly prevalent since the introduction of the concepts behind it approximately 20 years ago (Bateman and Organ, 1983). OCBs, or organizational citizenship behaviors, are activities that people of an organization do freely but which are neither prescribed nor rewarded in any way by their employer, included are activities that not only immediately benefit either the work group or the organization as a whole, but also contribute to the upkeep and maintenance of the social structure of the company. It is considered that citizenship behaviors might improve the operation of an organization by “greasing” its social machinery (Smith et al., 1983). Aditionally, citizenship behaviors can help the development of social capital in organizations (Jain and Cooper, 2012; Jain and Sharma, 2019) through the relation between OCBs exhibited by employees and their work groups. Many researchers have found evidence connecting stress and other mental health factors with OCB (Cialdini et al., 1982; Arunachalam, 2021; Bormann and Gellatly, 2021).

### Counterproductive work behavior

Counterproductive work behavior or CWB is defined as “intentional behavior that has the potential to disrupt or is driven to disrupt an organization and its stakeholders” (Hollinger and Clark, 1982; Spector and Fox, 2005). CWB includes oppositional behavior, antagonism (physical or verbal), inappropriate acts, sabotage, theft, and withdrawal, including absenteeism, tardiness, and abandoning a job. CWB is characterized by the criterion that all actions must be intentional and cannot be random. In other words, employees intentionally choose to disrupt the organization by acting in a specific manner. Numerous studies conducted over the past decade have highlighted not just the monetary cost of poor workplace behavior, but also its social and psychological effects on the organization (Hollinger and Clark, 1982; Penney and Spector, 2005; Simbula and Guglielmi, 2013; Mahd et al., 2018; Hudson and González-Gómez, 2021; Pati and Dhal, 2021). According to prior study, psychological detachment a form of depression (Liang and Hsieh, 2007), negative emotions and intelligence (Sahoo et al., 2022), occupational stress (Salami, 2010), burnout (Chiu and Tsai, 2010), and angry personality (Spector, 2011) likely have a significant influence in the prediction of CWBs. Although these studies have attempted to analyze CWB from a mental health perspective, a more in-depth examination is required after the covid period, which has caused a significant increase in mental health problems (Vadivel et al., 2021).

In contrast to OCB, which indicates a positive relationship between the individual and the organization, CWB suggests unfavorable employee conduct at work. CWB is “voluntary behavior that breaches significant organizational standards and affects the well-being of an organization, its members, or both” (Robinson and Bennett, 1997). CWB is a common but undesirable phenomenon in organizations. It has enormous effects on the performance of organizations, including decreased productivity, increased insurance costs, lost or destroyed property, and increased employee turnover (LeBlanc and Kelloway, 2002; Vigoda, 2002). Spector and Fox (2005) discovered evidence that emotions have a moderating role in the relationship between organizational restraints (a stressor) and CWB. Numerous studies conducted over the past decade have highlighted not just the economic cost of counterproductive workplace behavior, but also its social and psychological effects on the organization (Hollinger and Clark, 1983).

### Stress

Selye (1950) defined stress as a physiological response to a situation. According to Linden (2005), stress is a mechanism in which stressors (demands) evoke an effort at adjustment or settlement that, if failing, results in individual suffering. If the effort at adjustment or settlement is successful, the individual does not experience stress. Stress elicits physiological, behavioral, and cognitive responses. Chronic stress, as opposed to acute subjective or physiological activation, has the potential for the most detrimental health impacts. According to studies, stressed employees demonstrate poorer productivity, absenteeism, a larger frequency of accidents, worse morale, and increased interpersonal conflict with co-workers and superiors (Jain et al., 2011). Some occupations are extremely stressful due to their requirements, such as those in manufacturing industries like the automobile industry, which involves a variety of shift duties which causes work stress a continual noisy atmosphere which increases aggression (Alimohammadi et al., 2018; Subramaniam et al., 2019), and a regimented work schedule creating additional pressure (Omair et al., 2019) etc. Both Kim et al. (2015) and Akgunduz (2015) came to the conclusion that work stress negatively influences overall job performance. According to Tongchaiprasit and Ariyabuddhiphongs (2016), workplace stress can lead to a decline in job satisfaction as well as an increase in the desire to look for new employment.

### Anxiety

Anxiety is a generalized feeling of fear and apprehension. In contrast to fear and panic, the anxiety response pattern is a complex combination of unpleasant feelings and cognitions that is more future-focused and diffuse than fear. In addition to cognitive/subjective components, anxiety also includes physiological and behavioral components (Barlow, 2011). Employees generally fear about one’s jobs future aka job insecurity. In a longitudinal study, Spector and O’Connell (1994) discovered that frustration and anxiety are positive indicators of conflict. Spector and Jex (1998) discovered a correlation between conflict and a variety of unpleasant feelings, including depression, anxiety, and irritation. Anxiety is a negative emotion characterized by apprehension or concern. Anxiety is more closely associated with avoidant cognitive processes than with approach cognitive processes. Researchers discovered that anxiety is connected with Counterproductive Work Behavior in general (Fox and Spector, 1999; Fox et al., 2001). Chung et al. (2022) found evidence of anxiety mediating work behavior especially OCB and Wang et al. (2021) found evidence for leader workplace anxiety to be associated with employee OCB.

### Depression

Stress is almost often the cause of depression and anxiety and depression are the primary causes of mental illness, affecting millions of people on a global scale. Baranik and Eby (2016) demonstrated a connection between OCBs and employee depression, and research indicates that employees utilize OCBs to alter their emotional responses. According to prior studies, psychological detachment (depression) plays a crucial role in the prediction of CWBs following burnout (Liang and Hsieh, 2007). Sadness and despair, a form of depression, is believed to be associated with feeling of separation from the precipitating event (Beck et al., 1993; Assari and Lankarani, 2016; Lamis et al., 2018). Thus, occupational depression can lead to disengagement from the workplace, appearing as withdrawal behaviors or production deviation (Bauer and Spector, 2015).

The United States (US) economy’s emergence and importance was related to the auto industry as expressed by Henry Ford, the founder of the first auto firm, held ‘Freedom of mobility is the driving force behind human growth’ (Ford on why smart mobility is essential for smart cities | Automotive World, 2018). The importance of auto industry can not only be seen in the US, but also in India, which is the fifth-largest maker of automobiles, thus making the auto sector a significant economic force. The industry has an effect on the health, be it physical or mental, on its employees and specifically post the covid pandemic, it was noticed that there was a rise in extending a helping hand for the mental health programs among them (Purcell et al., 2021). The corporates were observed collaborating with non-profit partners to disperse funds, notably linked to providing mental health services, and supporting the transition of minority groups to a telehealth platform in order to meet healthcare demands during the pandemic. Thus, it can be seen that there was a significant rise in mental health factors in auto industries.

We try to investigate the unexplored conditions in which mental health may be likely to impede individuals from engaging in voluntary work behaviors post the covid pandemic. In order to accomplish this, we admit openly that mental health can impair discretionary work activities. Particularly, the loss of energy resources in the form of conservation of energy brought by covid is likely to diminish employees’ confidence in their ability to integrate normal job duties with volunteer work efforts, and it may also reduce their motivation to participate in such extra-role activities (Pooja et al., 2016; De Clercq and Belausteguigoitia, 2020b).

### Mental health in automobile sector

The automobile is one such Industry where work setting is tedious and requires increased physical presence than other industries. It is a highly competitive industry which continuously works to meet the demand supply chain cycle. Employees working there have to put in constant efforts to meet demand, undergo tremendous pressure to cope with the cycle. The tasks in the auto sector are among the most time- and stress-intensive. These circumstances have a negative impact on mental health. The sector’s mental health score in 2019 placed it in the bottom 10% of 19 industries (Manganello, 2019). According to Dr. Laura Hamill, an industrial and organizational psychologist, manufacturing industry employees face a unique combination of pressures that can contribute to high levels of stress, anxiety, and other mental health issues (Manganello, 2019). Also, as per Lang (2019) one out of every three engineers have felt that their mental health is deteriorating and that 22 per cent of the engineers have thought about self-harm or suicide. Addressing mental health issues at work not only helps in creating a better mental health platform but also increases the productivity of the organization.

In India research and studies on mental health outcome in automotive sector is limited, thus through our research we have humbly tried to understand the link between mental health and organizational behavior. India is the 5th largest manufacturer of automobiles in the world hence draws a special attention to the subject. The study location Chennai contributes 35 per cent of India’s automobile segment manufacturing.

## Theory and hypotheses

### Conservation of resources theory

COR theory (Hobfoll, 1989a) states that individuals who have sufficient resources to cope with the stress and challenges in their environment are less likely to experience stress. According to the theory people seek, preserve, foster, and protect their resources. It follows the idea that people cognitively overweight resource loss and underweight resource gain. The hypothesis states that micro aggressors like subtle distorted cognitions are real (Sue, 2010) and affect the individual. The theory claims that people are motivated to conserve and acquire resources. Resources might be thoughts, objects, or situations a person values. These materials’ worth depends on each person’s life experiences and circumstances. The theory posits that individuals who already own a greater number of resources have a greater capacity to acquire additional resources and a lower likelihood of experiencing a loss of resources that they already possess. This is attributable to the fact that those who already possess a number of resources have a stronger propensity to acquire more, particularly a predisposition to acquire more resources, the reason for this can be found in resource caravans put forward by Hobfoll et al. (2018). There are 4 principles and 3 corollaries to COR theory,

Principle

- Resource loss principle–the proportion of resource loss is higher in magnitude than resource gain
- Resource gain/investment principle–to recoup from resource loss an individual involves in resource gain or acquisition of resources
- Gain paradox principle – resource gain happens in salience to resource loss
- Desperation principle – occurrence of aggressive or irrational behavior because of extreme loss of resources

Corollary

- Individuals with greater resources are less vulnerable to resource loss and gain more resources
- Resource Loss Cycle–Because resource loss is more potent than resource gain and stress emerges when resources are lost, individuals and organizations have less resources to counterbalance resource loss with each stress spiral iteration, accelerating loss spirals
- Resource Gain Spirals–Resource gain spirals are weak and slow because resource gain is slower than resource loss

Conscientiousness, which is one of the OCB traits, is typically considered as a resource; Russell et al. (2017) discovered that persons with high levels of conscientiousness were more likely to utilize other resources in ways that were not always desirable. Conscientious persons experienced a faster decline in their emotional well-being than their counterparts who were less conscientious. In other words, conscientiousness is commonly considered a resource that enables individuals to manage other resources more effectively (Halbesleben et al., 2009; Ten Brummelhuis and Bakker, 2012), but it can also cause individuals to lose focus on broader performance goals.

### Hypothesis

Stress gives rise to anxiety and depression in the long run, stress is the basic precursor to any mental health conditions in an individual. We have considered stress and anxiety under one roof as the major explanation of the behavioral outcomes of the both is similar in nature.

Negatively impacted members of a project team could take a defensive stance and focus on maintaining the resources they still have (Hobfoll, 1989a), so they avoid behaviors that could further deplete those resources. Individuals on the team who are frustrated as a result may opt to neglect their duties if they have a greater degree of autonomy (Chiu and Tsai, 2010). Because OCB is awarded on a case-by-case basis, dissatisfied members of a team have the ability to decline or withhold OCB in order to protect their own resources (Perrewé and Zellars, 1999; Priesemuth and Taylor, 2016). Furthermore, given that feelings of resentment and anger are linked to job dissatisfaction (Eissa and Lester, 2018), a member of a team that is unhappy in their position may be less likely to exhibit OCB while they are going through a negative emotion. This is due to the fact that feelings of resentment and anger are correlated with dissatisfaction in one’s employment. In light of these things to take into account, we will now provide our preliminary hypothesis.

*Hypothesis 1*: Stress and Anxiety is negatively correlated with OCB.

The negative impacts of job stress are frequently explained by using a stress-emotional paradigm, which is used in existing research (Spector and Fox, 2005). Chen and Spector came to the conclusion that a person’s level of frustration directly correlates to the intensity of their hostile and aggressive behavior, and that anger is a common contributor to CWB Chen and Spector (1992). CWB is being negatively impacted by negative emotions, disruptive interpersonal behavior, vandalism, and attacks. In addition, studies have shown that those who experience a larger range of unpleasant emotions are more vulnerable to the effects that stresses have on them. Individuals who experience significant levels of negative emotions are more prone to incorrectly interpret stress as being intrusive in their responses to stressful events, which can lead to the development of CWB (Penney and Spector, 2005). Now we will provide the second hypothesis we possess.

*Hypothesis 2*: Stress and Anxiety is positively correlated with CWB.

Emotional regulation can be understood from the standpoint of conscience, in accordance with the theory of mood regulation developed by Tice and Bratslavsky (2000). People may put forth a concerted effort to intentionally enter, exit, and maintain either a pleasant or negative mood, and the majority of this effort is concentrated on overcoming unfavorable feelings like depression. Engaging in altruistic behavior is a healthy and adaptable way to work through these unfavorable feelings. Also assisting other people can be a good distraction since it forces people to concentrate not on their own negative feelings but on those of other people (Baranik and Eby, 2016). Thus, engaging in OCB’s can help an individual to overcome their negative moods and raise their happiness level. As per the addendum of the COR theory (Hobfoll et al., 2018) resource gain is spiraling in nature, and these gain spirals occur in saliency, under high loss conditions. As a result, the individual is highly motivated to rebuild or gain his lost resources, and he or she will work toward this goal even when faced with highly negative conditions. This builds up to our third hypothesis.

*Hypothesis 3*: Depression is positively correlated to OCB.

According to the findings of Liang and Hsieh (2007) psychological separation in the form of depression plays an important part in the prediction of CWBs that occur after burnout. As per Jones (1981) people who are mentally and emotionally drained are considerably more inclined to take breaks without permission and to cause harm to other people. Research reveals that emotionally exhausted professionals may adopt depression as a coping technique to avoid greater resource depletion. This is despite the fact that depression is typically associated with burnout (Cordes and Dougherty, 1993; Tomaka et al., 1993). The COR theory asserts that when individuals’ access to resources is severely limited, they are more likely to engage in defensive behaviors in an effort to forestall further resource depletion. With this argument we form our next hypothesis.

*Hypothesis 4*: Depression is negatively correlated with CWB.

Leveraging on COR theory (Hobfoll, 1989b, 2001; Hobfoll et al., 2018) as a paradigm, we seek to explore the relationship between mental health, OCB and CWB, we propose that mental health might be a significant factor in impacting organizational behavior.

## Method

### Participants and setting

The study was carried out at an automobile components manufacturer organization in Chennai, India. The participants consisted of 51 white collar and 43 Blue collar permanent employees between the ages of 21 and 60 years. The sample size for the research was 94 as the auto sector unit consisted of population of around 600 employees and a random sampling technique without replacement was done to select the subjects, only permanent employees of the organization were selected for the survey, also the said population fulfills the criteria to perform significant statistical calculations and to assert enough confidence intervals of the population. Informed consent was obtained from the participants through written format. Seventy percent of the respondents were between the ages of 21 and 30, while 14 % were between 31 and 40 and the rest between 41 and 60 years. Details were obtained from both white collar (54 per cent) and blue collar (46 per cent) personnel. As primarily a manufacturing organization, only 9 % of the population was female thus contributing more than 90 percent of the study population to be male. Eighty percent of the population was married, while the remaining 20 % were unmarried or single. The income of the participants ranged from 20 to 30 thousand (25 per cent), 30–50 thousand (43 per cent), 50–80 thousand (14 per cent), 80 thousand to 1 lac (7 per cent), and 1 lac and above (11 per cent). The company provided all the safety measures and guidelines for the employees to work smoothly. Proper social distancing, masking, wearing of gloves, goggles in shopfloor, caps, uniforms were maintained.

### Procedure

The study was conducted right after the third covid partial lockdown period was lifted in Tamil Nadu (COVID-19 pandemic in Tamil Nadu, 2022) from March to April 2022. The employees in the study belonged to automobile industry and thus had to commute to office on a daily basis with severe safety protocols. Participants were chosen at random, and there were no stand-ins allowed. Workers from both white- and blue-collar backgrounds were included in the sample. The survey was carried out using the old-fashioned method of paper and pen. After a period of 30 min, the researcher went back to the location where he or she had initially delivered the questionnaires in order to collect the completed questionnaires from the white-collar employees who were working at their respective work stations. The shift operators completed the questionnaire during a break of 30 min that they received from their pressing and forging operations. The following scales were used in the survey: Depression Anxiety Stress Scale (DASS-21) developed by Lovibond and Lovibond (1995), Organizational Citizenship Behavior-Checklist (OCB-C 20) (Fox and Spector, 2009) and Counterproductive Work Behavior–checklist (CWB-C 10) (Spector and Fox, 2010) both developed by Paul Spector and Suzy Fox. The Statistical Package for the Social Sciences (SPSS 26) was employed in order to carry out the calculations for the statistical analysis of the data. Spearman’s Rho was used for calculating the correlation as it is a more appropriate measurement when an ordinal scale is used.

## Results and discussion

The correlation between stress, anxiety, depression with OCB and CWB through the lens of COR theory are discussed in results and discussion. After analyzing the information using the SPSS software the following results were obtained.

From Table 1 it can be clearly seen that employees in the automobile sector have experienced higher anxiety and OCB among all other aspects and have experienced lowest stress and CWB in their organization. The depression levels of the employees were found to be moderate.

**Table 1:** Displays the levels of stress, anxiety, depression, OCB and CWB experienced by the employees.

	Low	Medium	High
Stress	46	41	7
Anxiety	15	–	79
Depression	35	39	20
OCB	–	39	55
CWB	89	5	–

According to Table 2, OCB shows a significant positive correlation with stress and anxiety at the 0.01 level and with depression at the 0.05 level, suggesting that a rise in the independent variable causes a rise in the dependent variable. Thus, we reject our hypotheses 1 and accept hypothesis 3 as the variables are positively connected with OCB. At the 0.05 significance level, a negative correlation was found between the variables CWB with stress and anxiety, indicating that an increase in one variable leads to a reduction in the other. There was no link between CWB and depression. Therefore, we reject hypotheses 2 and 4. These data underwent additional analysis by using the regression approach in order to determine their respective explanatory values.

**Table 2:** Displays the SPSS analysis of the correlation between the variables.

	Stress	Anxiety	Depression	OCB	CWB
Stress	1	–	–	–	–
Anxiety	0.768**	1	–	–	–
Depression	0.475**	0.535**	1	–	–
OCB	0.368**	0.268**	0.204*	1	–
CWB	−0.206**	−0.298**	0.019	−0.149	1
Mean	15.55	15.91	14.39	67.94	36.43
SD	7.242	8.172	8.704	16.893	9.803

*N* = 93; ^*^p < 0.05 level; ^**^p < 0.01 level.

**Figure 1:**
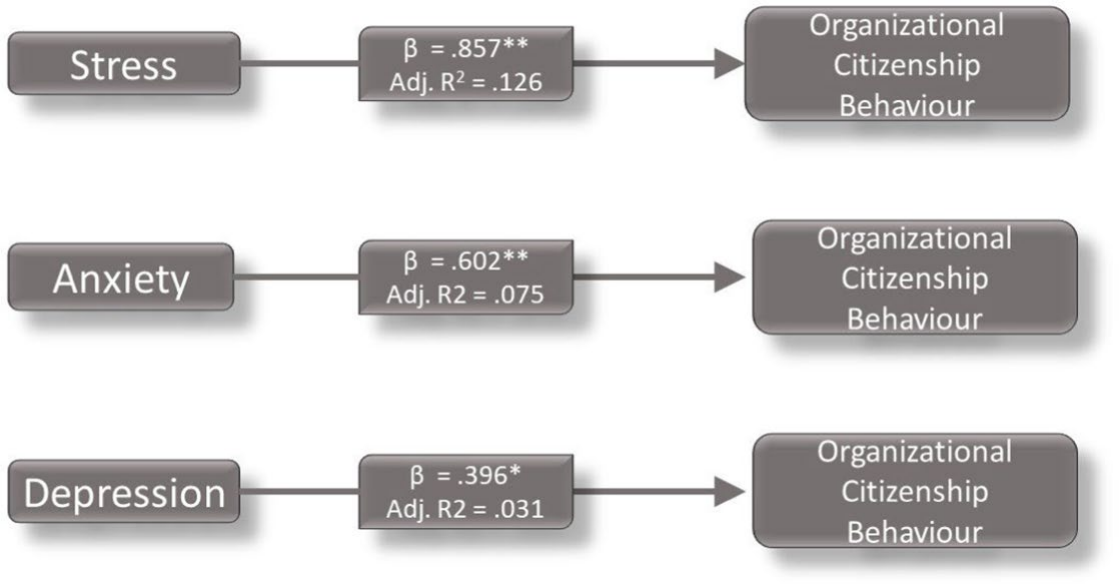
Regression results (** significant at 0.01 level, * significant at 0.05 level).

The results of the regression analysis of the data with the OCB dependent variable are presented in Figure 1. Their regression results also suggest that the data are significant with stress and anxiety at the level of 0.01 and with depression at the level of 0.05 which explains the variables effect at 12 per cent, 7 per cent and 3 per cent, respectively. There were no significant results of the regression results for CWB. We found that the results of the data provided evidenced against the aforementioned hypothesis; accordingly, we would like to analyze these results through the lens of COR theory and explain the resulting shifts in our hypotheses.

## Discussion

The core of the COR theory (Hobfoll, 1989a) is the idea that people will go to great lengths to preserve the things they care about most, whether it be their health, their family, their sense of well-being, sense of self-worth, etc., the theory revolves around stress and its outcomes. Due to the fact that we found that there was a positive connection between stress and OCB and anxiety with OCB, which was counter to what was stated by the existing body of research but was supported by the SPSS data, we were forced to reject our first hypothesis. When there is a resource loss, such as stress in our situation, there will be a resource gain happening in silence, such as OCB, and this conclusion is quite startling and novel as this explains a negative effect on each other. Now how do we explain the occurrence of this phenomenon which is statistically evident? This can be explained by the *Gain Paradox principle* of COR theory which indicates that resource gain increases in salience with resource loss. So, we can say that employees feeling stressed or anxious are more likely to engage in OCB in an effort to keep morale high to maintain and gain in their resources of emotional well-being. These findings are also consistent with the negative state relief model proposed by Cialdini and colleagues (Cialdini and Kenrick, 1976; Cialdini et al., 1982), which suggests that altruistic actions are driven by the wish to alleviate one’s own feelings of unhappiness or dissatisfaction. As a result, it is reasonable to believe that engaging in any form of constructive behavior (like OCBs) can help alleviate stress (Jain and Cooper, 2012).

Furthermore, in the study, it was shown that stress and anxiety had a negative correlation to CWB as against our hypothesis number two. Though their regression results were not a good predictor of each other, they still stand statistically evident for correlation. This action of negative impact of stress and anxiety on CWB is new and the explanation of it can be found under the COR theory umbrella in accordance with the fourth principle of the theory, *the Desperation Principle*. The principle states that when an individual’s resources are exhausted or outstretched, they enter a defensive mode to preserve themselves by becoming defensive, aggressive, or irrational. Thus, employees exhibit CWB (an aggressive/defensive state) when their resource (stress) is lost. These contradictory results of Stress with OCB and CWB having positive and negative cross over with each other can be explained by Bakker et al. (2009), Westman (2016), and Hobfoll et al. (2018) crossover model, and that a part of the study population influenced each other or team members (Bakker et al., 2009; Ten Brummelhuis and Bakker, 2012).

A positive link between OCB and depression was established corroborating the earlier researches (Tice and Bratslavsky, 2000; Baranik and Eby, 2016) and accepting our hypothesis number four. Depression is a more severe symptom than stress and anxiety together, and it is made worse by high-negative events. These negative events or uneventful circumstances in a person’s life causes immense depression, making the individual to either come out of it or go with it. Trying to come out or gain from an uneventful situation is a form of resource gain according to COR theory and as per the theories corollary 3, *Resource Gain Spirals* (Hobfoll et al., 2018), claims that an individual’s desire to generate a resource gain will increase when a resource loss happens, and that this motivation will be at a high level when situations of extreme stress are prevalent. Depression being a highly stressful condition can make an individual to gain resources, thus a person will slowly engage in resource-gaining behaviors such as OCB in order to alleviate the pressure (like depression) they are under.

### Implications

Mental health, comprised of stress, anxiety, and depression, is an essential component of one’s personal and professional life, and its effects on organizational behavior, notably citizenship and counterproductive behavior, are significant. This study of the relationship between mental health and organizational behavior as a predictor of OCB and CWB has significant organizational consequences. The COR theory adds to its significance by providing multiple explanations for the causal effect. Novel in its practice, the study aims to explain a phenomenon *via* the lens of the evolving COR theory, which has excellent explanatory power for important outcomes that do not fit the standard framework.

### Limitations

The study was exclusively carried out in an Automobile sector organization which contributes to 7 per cent of India’s GDP. The sample population was quiet young (averaging in 20s) and majorly male (more than 90 per cent) which might be a reason for having a different result from suggested hypothesis. Also, the study did not take into consideration the socio-demographic details for analysis as we wanted to first analyze the link of the COR theory with the variables majorly. The study also did not consider the role of the employees within the organization for comparison, which can be taken up for future studies, as many abusive supervision (Hussain and Sia, 2017) studies have revealed a lot of workplace deviance. The context of this research is extremely important to understanding how these results should be interpreted. The socially detrimental variables need to be incorporated in the study’s continuation because working in a manufacturing unit is inherently stressful due to the physical demands, lengthy travel hours, and the existing covid environment. The study was done right immediately after the third lockdown period thus the voluntary participation of the employees was comparatively less considering the safety protocols. The fact that the OCB self-report measure is entirely dependent on self-reporting is another key flaw in the instrument. Due to the fact that the scope of this study is restricted to the automotive industry, we are unable to generalize the findings to apply to any other industries. When conducting future research with variables that are comparable, it will be necessary to draw comparison samples from populations that are demographically and geographically varied.

## Conclusion

The present study investigated the impact of stress, anxiety and depression on OCB and CWB of an automobile sector employee in India. It found out that direct mental health conditions have a positive and negative link on employee work behaviors. OCB was seen to have a positive relationship with stress, anxiety and depression and CWB to have a negative relationship with stress and anxiety. The results were analyzed using the COR theories principles and corollaries and we found out why an increase in stress and anxiety also causes an increase in OCB according to the gain principle. Similarly, the expected inverted relationship between CWB and stress and anxiety were explained through the theories desperation principle. The COR theory gave us a great explanatory power on the causes and effects of behavior in an organization having different outcomes for a similar phenomenon. Also, the study found out that the mental health conditions in an organization is important and needs to be addressed specifically as they have a direct and indirect effect on employee behaviors at organizations. Outsourced mental health services, or an inhouse employee counseling can help them in managing their day-to-day stress and anxiety issues as prolonged onset of stress and anxiety leads to chronic depression (Heilig, 2004) affecting the functioning of the individual. Employee mentoring programs and additional fringe benefits can also help lessen the mental health consequences at work as they have a complementary effect on the work. Mental Health assessment on industries in India needs more research on a large scale to help and make the workplace a better place to be.

## Data availability statement

The raw data supporting the conclusions of this article will be made available by the authors, without undue reservation.

## Ethics statement

Ethical review and approval was not required for the study on human participants in accordance with the local legislation and institutional requirements. The patients/participants provided their written informed consent to participate in this study.

## Author contributions

AS is a research scholar who has conceptualized, written, and developed the paper. MB is the research supervisor and has supervised the paper. All authors contributed to the article and approved the submitted version.

## Conflict of interest

The authors declare that the research was conducted in the absence of any commercial or financial relationships that could be construed as a potential conflict of interest.

## Publisher’s note

All claims expressed in this article are solely those of the authors and do not necessarily represent those of their affiliated organizations, or those of the publisher, the editors and the reviewers. Any product that may be evaluated in this article, or claim that may be made by its manufacturer, is not guaranteed or endorsed by the publisher.
